# Dahl (S x R) Congenic Strain Analysis Confirms and Defines a Chromosome 5 Female-Specific Blood Pressure Quantitative Trait Locus to <7 Mbp

**DOI:** 10.1371/journal.pone.0042214

**Published:** 2012-07-30

**Authors:** Victoria L. M. Herrera, Khristine A. Pasion, Ann Marie Moran, Nelson Ruiz-Opazo

**Affiliations:** Section of Cardiovascular Medicine, Department of Medicine, and Whitaker Cardiovascular Institute, Boston University School of Medicine, Boston, Massachusetts, United States of America; Leibniz-Institute for Arteriosclerosis Research at the University Muenster, Germany

## Abstract

The detection of multiple sex-specific blood pressure (BP) quantitative trait loci (QTLs) in independent total genome analyses of F2 (Dahl S x R)-intercross male and female rat cohorts confirms clinical observations of sex-specific disease cause and response to treatment among hypertensive patients, and mandate the identification of sex-specific hypertension genes/mechanisms. We developed and studied two congenic strains, S.R5A and S.R5B introgressing Dahl R-chromosome 5 segments into Dahl S chromosome 5 region spanning putative *BP-f1* and *BP-f2* QTLs. Radiotelemetric non-stressed 24-hour BP analysis at four weeks post-high salt diet (8% NaCl) challenge, identified only S.R5B congenic rats with lower SBP (−26.5 mmHg, *P = *0.002), DBP (−23.7 mmHg, *P = *0.004) and MAP (−25.1 mmHg, *P = *0.002) compared with Dahl S female controls at four months of age confirming *BP-f1* but not *BP-f2* QTL on rat chromosome 5. The S.R5B congenic segment did not affect pulse pressure and relative heart weight indicating that the gene underlying *BP-f1* does not influence arterial stiffness and cardiac hypertrophy. The results of our congenic analysis narrowed *BP-f1* to chromosome 5 coordinates 134.9–141.5 Mbp setting up the basis for further fine mapping of *BP-f1* and eventual identification of the specific gene variant accounting for *BP-f1* effect on blood pressure.

## Introduction

Polygenic (essential) hypertension is a leading risk factor for heart disease, stroke and renal failure [1]. Due to its complex inheritance, the genetic determinants of susceptibility to hypertension and its end organ diseases in humans remain to be fully elucidated [2]–[4]. Hypertension is further compounded by phenotype variation due to its relatively late onset, variable disease course and target organ complications, sex-specific differences and emerging impact of gestational environmental factors. This multi-faceted complexity has made elucidation of hypertension susceptibility genes challenging. Moreover, given differential responses to therapy and end-organ disease outcomes, it becomes apparent that hypertension genes are likely hypertension subtype-specific, and modified by diet and developmental programming, which are not accounted for in reported multi-center genetic cohort analyses [5]–[7].

Animal models of polygenic hypertension offer the ability to eliminate major confounding from diet and developmental programming and conduct controlled genetic experiments to localize BP QTLs on their genomes [1], [8]. Our earlier studies in F2-intercross male and female populations derived from Dahl salt-resistant (Dahl R/jr^HS^) and Dahl salt-sensitive (Dahl S/jr^HS^) hypertensive inbred rat lines established sex-specific quantitative trait loci for BP and end organ disease [9]. We detected a female-specific BP QTL region on chromosome 5 (100–140 Mbp) with significant linkage [9]. The initial analysis suggested either the possible presence of two closely linked BP QTLs or that the position for this QTL was not well defined [10]. The present study was undertaking to 1) confirm the presence of one or two BP QTLs in this region, and 2) delimit more precisely the chromosomal region (s) harboring this BP QTL.

## Results

**Figure 1:**
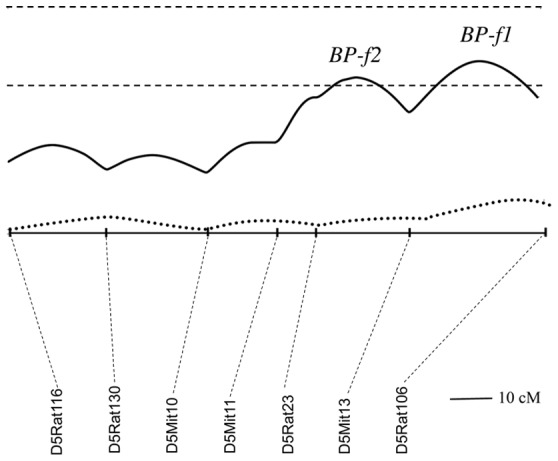
Chromosome 5 blood pressure (BP) QTLs in male and female F2 [Dahl S x R]-intercross rats. Chromosome 5 was analyzed by interval mapping in male and female F2 [Dahl S x R]-intercross rat populations (QTX Map Manager) [10]. Orientation of chromosome: left → right starting from lowest Mbp. Horizontal dashed lines mark LOD values for significance of linkage, from top to bottom: highly significant LOD ≥4.9 and significant LOD ≥3.2. LOD in female F2 [Dahl S x R]-intercross rats (black solid line); LOD in male F2 [Dahl S x R]-intercross rats (dotted line).

**Figure 2:**
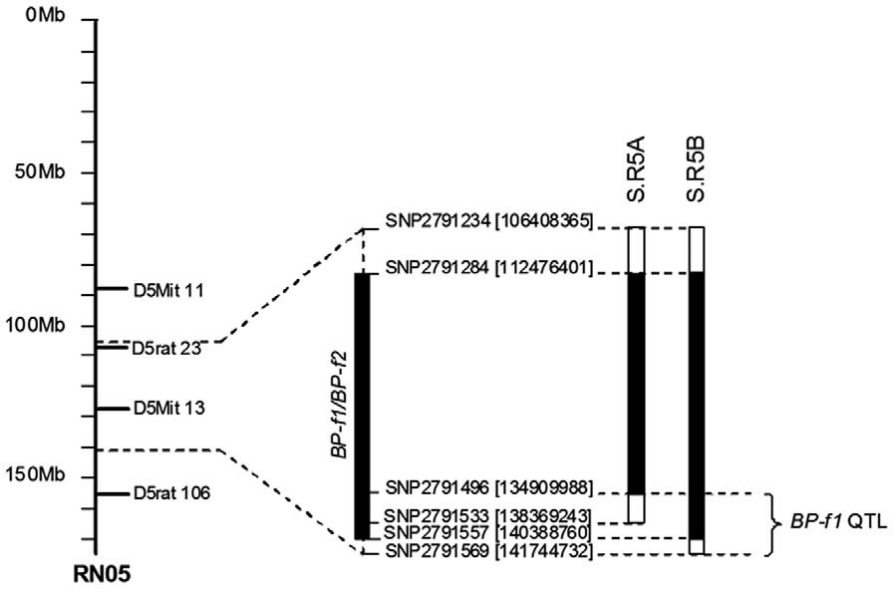
Congenic analysis of *BP-f1* QTL region on chromosome 5. On the left of the figure is shown the relevant region of the physical map of rat chromosome 5. Values in parenthesis next to the SNP names denote physical locations in base pairs. The mapped *BP-f1* QTL region of approximately 6 Mbp is noted (to the right). Congenic strains are shown as solid bars (representing the Dahl R introgressed fragments) flanked by open bars (representing the putative regions of recombination).

Our previous linkage study delineated the potential existence of two closely linked female-specific BP QTLs on chromosome 5, *BP-f1 and BP-f2* (Figure 1) [9]. To substantiate the existence of one or two BP QTLs in this region, we transferred two Dahl R chromosomal segments spanning the *BP-f1/BP-f2* QTL region onto the Dahl S genetic background. For this purpose we screened 300 BC1 (back-cross 1) male subjects for recombinants carrying the Dahl R chromosome 5 *BP-f1* and/or *BP-f2* QTL regions with informative markers. We identified two congenic fragments spanning the region (shown in Figure 2). Each congenic segment (S.R5A and S.R5B) was carried at least by one potential male breeder. We successfully implemented a “speed congenic” strategy towards the development of highly inbred S.R5A and S.R5B (Figure 2) congenic lines. Back-crosses were performed up to BC6 at which level we established homozygous congenic lines for blood pressure measurements. At BC6 S.R5A was >99.85% of Dahl S genetic background and S.R5B >99.75% of Dahl S genetic background.

Congenic analysis of the chromosome 5 region spanning putative *BP-f1* and/or *BP-f2* QTLs (Figure 2) substantiated the existence of only one BP QTL in this region as demonstrated by the significantly lower systolic, diastolic and mean blood pressures exhibited by SR.5B rats compared with Dahl S controls (from now on called *BP-f1*, Table 1). Introgression of the S.R5B congenic region onto the Dahl S genetic background lowers systolic blood pressure by 26.5 mmHg (Table 1). Blood pressure in S.R5A congenic rats did not differ from Dahl S controls demonstrating absence of genes affecting blood pressure in this chromosomal region. Moreover, the blood pressure of the S.R5A rats containing the S.R5A congenic segment was equivalent to the blood pressure measured in Dahl S controls delimiting the chromosomal region to <7 Mbp (134.9–141.5 Mbp) that contains the gene underlying *BP-f1*. No difference in pulse pressure between SR.5B and Dahl S control rats was detected (Table 1) suggesting that the gene underlying *BP-f1* QTL might not affect arterial stiffness. Similarly, no differences in RHW (Table 1) were detected between the congenics and Dahl S controls implying absence of genetic effects on cardiac hypertrophy within this chromosomal region.

**Table 1:** Effects of female rat chromosome 5 congenic strains on blood pressure, pulse pressure and relative heart weight.

Strain	n		Δ	*P*
***SBP***				
Dahl S	9	194.4±6.2		
S.R5A	10	185.9±3.8	8.5	>0.2
S.R5B	8	167.9±3.2	26.5	**0.002**
***DBP***				
Dahl S	9	139.7±5.8		
S.R5A	10	131.9±3.6	7.8	>0.2
S.R5B	8	116.0±3.3	23.7	**0.004**
***MAP***				
Dahl S	9	165.9±5.9		
S.R5A	10	157.7±3.7	8.2	>0.2
S.R5B	8	140.8±3.1	25.1	**0.002**
***PP***				
Dahl S	9	54.74±0.8		
S.R5A	10	54.11±0.4	0.63	>0.5
S.R5B	8	52.03±1.4	2.71	>0.1
***RHW***				
Dahl S	9	3.316±0.14		
S.R5A	10	2.946±0.05	0.380	>0.1
S.R5B	8	3.135±0.30	0.181	>0.6

Values are means ± standard error of the means; n, number of animals; Δ, difference between Dahl S and congenic values; SBP, systolic blood pressure in mmHg; DBP, diastolic blood pressure in mmHg; MAP, mean arterial pressure in mmHg; PP, pulse pressure; RHW, relative heart weight (ratio of heart weight to body weight multiplied by 1000); *P*, one-way ANOVA followed by Holm-Sidak’s test for multiple comparisons.

## Discussion

Our initial linkage study results in the female F2(Dahl S x R) intercross population showed suggestive evidence for two BP QTLs in chromosome 5 100–140 Mbp region by the apparent presence of two confidence interval peaks for this QTL location, *BP-f1* and *BP-f2* [9]. Results obtained in the present study confirmed the existence of a single chromosome 5 BP QTL, *BP-f1*, but not *BP-f2*, implying that the original confidence interval data was indicating that the position for *BP-f1* was not well defined instead. Our congenic analysis localizes the *BP-f1* QTL between 134.9–141.5 Mbp (Figure 2) on chromosome 5. Importantly, inspection of the few genome scans for BP QTLs performed on rat female subjects [9], [11]–[13] reveal that the chromosome 5 region spanning *BP-f1* has also been linked to blood pressure in a linkage study performed in Wild rats (Rattus norvegicus) using the SHR rat as contrasting hypertensive strain [11] and in a (Dahl S x Brown Norway)- female intercross cohort [12]. In parallel, the human syntenic region (chromosome 1 41–48 Mbp) corresponding to *BP-f1* in the rat has also been associated with blood pressure in humans [14].

Two closely linked interactive BP QTLs have been reported on chromosome 5 in male congenic Dahl S rats introgressed with genomic fragments from the LEW strain [15], [16]. QTL1 was localized between coordinates 124.08–134.97 Mbp and QTL2 between coordinates 116.97–121.64 Mbp [15], [16]. However, a related study [17] using two congenic lines developed by Garrett and Rapp (2002) [15], (S.LEW(5) ×4 and S.LEW(5) ×6), detected a single BP QTL in this region mapping to 134.8–139.9 Mbp. Based on additional functional analysis Roman et al (2006) [17] provides supportive evidence for the CYP4A gene cluster (CYP4A8, CYP4A2, CYP4A3 and CYP4A1) localized between 135.55–135.92 Mbp on chromosome 5 as candidates genes underlying this QTL [17]. The discordant results have been tentatively explained by differences in salt-loading with 2% NaCl containing diet [15] versus 8% NaCl containing diet [17]. However, the onset of the high salt challenge was also different, juvenile rats at 6 weeks of age [15] and adult rats at 9 weeks of age [17] respectively which could explain the different results. Nevertheless, the existence of multiple BP QTLs in this chromosome 5 region affecting salt-sensitive hypertension in Dahl S male rats and putative genes underlying these QTLs remains to be elucidated.

Our chromosome 5 female-specific *BP-f1* QTL localizes between 134.9–141.5 Mbp, region that overlaps with the QTL defined by Roman et al (2006) (134.8–139.9 Mbp) [17]. Thus, our region could include the Dahl R CYP4A gene cluster as well (135.55–135.92 Mbp). However, the CYP4A gene cluster resides within our putative region of recombination (134.90–138.37 Mbp, Figure 2) making uncertain if the S.R5B congenic segment had retained the Dahl R CYP4A alleles. Additional fine mapping of this region in our S.R5A congenic line could determine if the CYPA4 gene cluster will remain as candidate genes for our female-specific *BP-f1* QTL. Nevertheless, it is quite possible that the gene underlying *BP-f1* QTL differs from the genes underlying the BP QTLs detected in males [15]–[17] considering the differential use of contrasting normotensive strains, LEW [15]–[17] and Dahl R in our studies. Edn2 (endothelin-2) at 140.75 Mbp is noted as a potential candidate gene for *BP-f1* because of its role in blood pressure regulation [14], [18]. Notably, Edn2 has been associated with human essential hypertension in a Caucasian population of European origin [14], although specific functional variants have not been elucidated accounting for the susceptibility to high blood pressure. Concordance of rat and human studies supports the hypothesis that Edn2 is a candidate hypertension gene in females.

In conclusion, our study demonstrates the existence of a single BP QTL on chromosome 5 affecting blood pressure in Dahl S female rats. The successful trapping of the chromosome 5 *BP-f1* QTL present in S.R5B congenic line form the basis to further fine map this QTL region to <1 Mbp via sub-strain construction and eventually identify the specific gene variant accounting for *BP-f1* QTL. Increasing experimental evidence show that the genes underlying essential hypertension are different between sexes [9], [11], [13], [19]–[22], thus our findings provide further compelling evidence to prioritize the elucidation of genetic mechanisms in female hypertension as an *a priori* basis for novel prevention and intervention strategies for the female population.

## Materials and Methods

### Strains

This study was performed in strict accordance with the recommendations in the Guide for the Care and Use of Laboratory Animals of the National Institutes of Health. The protocol was approved by the Committee on the Ethics of Animal Experiments of Boston University School of Medicine (Permit Number: AN-14966). All surgery was performed under sodium pentobarbital anesthesia, and every effort was made to minimize suffering. All rats utilized in this study were bred in-house. Inbred Dahl S/jrHsd and Dahl R/jrHsd rats were obtained from Harlan (Indianapolis, Indiana). We transferred several Dahl R chromosomal segments spanning *BP-f1* onto the Dahl S genetic background. We implemented a “speed congenic” strategy [23], [24] to develop the different congenic lines. For this purpose we first produced a (Dahl S x Dahl R) F1 progeny followed by generation of an F1 x Dahl S backcross (BC1) population. We selected *BP-f1* “carriers” from 300 BC1 subjects by genotyping the BC1 male progeny with flanking markers of the chromosomal segments planned to be transferred. For S.R5A; sr heterozygous at SNP2791284 and SNP2791496, and ss homozygous at nearby flanking markers, i.e. SNP2791234 and SNP2791553. For S.R5B; sr heterozygous at SNP2791557 and SNP2791284, and ss homozygous at nearby flanking markers, i.e. SNP2791234 and SNP2791569. We then produced 20 BC2 male subjects per congenic line and proceeded to screen subjects with 85 informative SNPs. One “best” male breeder per congenic line was chosen to continue with the inbreeding program. Back-crosses were performed up to BC6 at which level we established homozygous congenic lines for blood pressure measurements. At BC6 S.R5A was >99.85% of Dahl S genetic background and S.R5B >99.75% of Dahl S genetic background.

### Markers

We selected the following single nucleotide polymorphisms (SNPs) for congenic rat development from the rat genome data base (RGD): markers for S.R5A and S.R5B congenic fragments; SNP2791234, SNP2791284, SNP2791496, SNP2791553, SNP2791557, SNP2791569. SNPs for implementation of “speed congenic” strategy, chr1: SNP2783361, SNP2783513, SNP2783573, SNP2783925, SNP2784073, SNP2784200, SNP2784723, SNP2784895, SNP2785046; chr2: SNP2785301, SNP2785499, SNP2785693, SNP2785860, SNP2786134, SNP2786276, SNP2786350, SNP2786619, SNP2786811, SNP2786979, SNP2787226; chr3: SNP2787599, SNP2787751, SNP2787947, SNP2788108, SNP2788217, SNP2788416; chr4: SNP2789191, SNP2789416, SNP2789717, SNP2789952, SNP2790223, chr5: SNP2790571, SNP2790733, SNP2790960, SNP2791234, SNP2791496, SNP2791711, SNP2791834; chr6: SNP2792065, SNP2792467, SNP2792754; chr7: SNP2793338, SNP2793565, SNP2793757, SNP2793904; chr8: SNP2794281, SNP2794450, SNP2794721, SNP2794865; chr9: SNP2795738, SNP2795947; chr10: SNP2796278, SNP2796474, SNP2796739, SNP2796966; chr11: SNP2797258, SNP2797443, SNP2797742; chr12: SNP2797924, SNP2798115; chr13: SNP2798475, SNP2798659, SNP2798785, SNP2798926; chr14: SNP2799254, SNP2799430, SNP2799825; chr15: SNP2800105, SNP2800195; chr16: SNP2800810, SNP2801108; chr17: SNP2801413, SNP2801584, SNP2801868, SNP2801948; chr18: SNP2802358, SNP2802507, SNP2802706; chr19: SNP2802997, SNP2803270; chr20: SNP2803540, SNP2803747; chrX: SNP2804065, SNP2804185, SNP2804233.

### Genotyping

DNA was extracted from tail biopsies using the QIAamp Tissue Kit (Qiagen, Valencia, CA). SNP genotyping was carried out on an Applied Biosystems 7900 Real-Time PCR System. All SNP assays (TaqMan assays) were procured from Applied Biosystems and were validated in our laboratory.

### Blood Pressure Measurements

Animals were maintained on a Harlan 2018 rodent chow (Harlan Teklad, Madison WI) containing 0.23% NaCl from weaning until the high salt diet begun at 12 weeks of age. The food pellets and water were made available ad lib. Blood pressure (BP) was measured essentially as described [9], [25] using intra-aortic abdominal radiotelemetric implants (DATASCIENCE) obtaining non-stressed blood pressure measurements taking the average over ten-seconds every 5 minutes for 24 hours [9], [25]. Systolic (SBP), diastolic (DBP) and mean arterial pressures (MAP) were obtained along with heart rate and activity. The protocol for the congenic and control female rats was as follows: implant surgery at 10 weeks of age; after 12 days, baseline BP levels were obtained. The high salt (8% NaCl) challenge was initiated at 12 weeks of age and maintained for four weeks for all rats as described [9], [25]. BP values used for phenotype comparison were the averages obtained for the last weekend of the salt loading from Friday-Monday with minimal entry to BP room ascertaining non-stress BP.

### Statistical Analyses

We performed one-way ANOVA followed by all pairwise multiple comparisons using the Holm-Sidak test for blood pressure and relative heart weight (RHW) as indicated per experimental comparison. Relative heart weight was calculated as the ratio of heart weight to body weight multiplied by a factor of 1000.
